# Circulating tumor cells with karyotyping as a novel biomarker for diagnosis and treatment of nasopharyngeal carcinoma

**DOI:** 10.1186/s12885-018-5034-x

**Published:** 2018-11-19

**Authors:** Jing Zhang, Huashan Shi, Tingting Jiang, Zhe Liu, Peter P. Lin, Nianyong Chen

**Affiliations:** 1 Department of Radiation Oncology, Cancer Center and State Key Laboratory of Biotherapy, West China Hospital, Sichuan University and Collaborative Innovation Center, 37 GuoXueXiang, Wuhou District, Chengdu, 610041 Sichuan China; 2 Cytelligen, San Diego, California USA

**Keywords:** Nasopharyngeal carcinoma, Circulating tumor cells, Karyotyping, SE-iFISH, Biomarker

## Abstract

**Background:**

Circulating tumor cells (CTCs) have been considered great clinical significance in various cancers. However, it remains unknown that how is the role of CTCs in patients with nasopharyngeal carcinoma (NPC). We investigated the value of CTCs enumeration and karyotyping in NPC.

**Methods:**

In the present study, we applied integrated subtraction enrichment and immunostaining-fluorescence in situ hybridization (SE-iFISH) automatic testing system to detect and characterize CTCs of NPC patients. Enumeration and aneuploidy of chromosome 8 in CTCs were examined in various stages of patients with NPC. The changes of CTCs number and karyotyping post to chemotherapy were investigated in NPC.

**Results:**

CTCs were detected by SE-iFISH in 46 out of 50 pre-treatment NPC patients, and performed a positive rate of 92.0%. No significant association was found between disease staging and CTCs detection rate. CTCs number constantly increased with TNM stage rising (from stage II to stage IV) no matter in newly diagnosed patients without distant metastasis (M0) and relapsed or distant metastatic patients. The number of CTCs decreased after treatment in patients with partial response (PR), while increased in patients with progressive disease or stable disease (PD/SD). More interestingly, CTCs karyotyping indicated that aneuploidy of chromosome 8 in CTCs was dramatically related to chemotherapeutic efficacy in NPC. Positive correlation was found between CTCs count and plasma EBV DNA level of NPC patients.

**Conclusions:**

CTCs could be detected in various stages of NPC patients using SE-iFISH. CTCs number could indicate the severity degree of disease in NPC. Dynamically monitoring the variations in CTCs number may predict chemotherapy efficacy during treatment. CTCs karyotyping is related to the sensibility of chemotherapy and drug resistance, and karyotyping of CTCs might predict therapeutic efficacy and evaluate chemo-resistance in NPC. CTCs could be used as a monitoring indicator in the fields of treatment, diagnosis and follow-up of NPC.

## Background

Nasopharyngeal carcinoma (NPC) is a disease with low prevalence in large parts of the world, while it is notable for its high incidence in South China and South Asia. In China, there were an estimated 60,600 new cases of NPC and 34,100 deaths in 2015 [1–3]. NPC is an endemic carcinoma, which is different from other head and neck cancers on account of its epidemiology, histopathology, and clinical characteristics. There are always no symptoms until the cancer has metastasized to other sites of the body such as the neck. More than 70% of newly diagnosed cases of NPC are classified as locoregionally advanced disease [4]. Radiation therapy (RT) has been the primary treatment modality for NPC based on its inherent anatomic constraints and high sensitivity to irradiation. Previous studies have showed that concurrent chemoradiotherapy (CCRT) was more effective than RT alone for locoregionally advanced NPC, and became the standard treatment for those patients. Recently, induction chemotherapy (IndCT) followed CCRT significantly improved failure-free survival in locoregionally advanced NPC [5, 6].

Despite the survival rates significantly enhanced from modern treatment techniques, however, treatment failure is still due to distant metastasis, relapse, or both, leading 20 to 30% of patients′ death. Clinicopathologic characteristics such as local invasion (T stage), regional extension (N stage) have been revealed to correlate with the occurrence of relapse and metastasis, and TNM stage is a reliable predictive factor of prognosis in NPC [7]. Clinically, diagnosis and TNM classification for NPC mainly rely on imaging technique such as magnetic resonance imaging (MRI), in addition, plasma Epstein-Barr virus (EBV) DNA level is useful for monitoring and predicting treatment outcome of NPC. But they are often failed to detect tumor lesions in early stage and assess therapeutic response from NPC in time due to the hysteresis and instability. Therefore, there is an urgent need for a novel biomarker to early diagnosis, predicting therapy efficacy and identifying patients at high risk of treatment failure, who could be treated with an intensive treatment strategy in time to improve survival of NPC [8, 9].

Circulating tumor cells (CTCs) are tumor cells disseminated from primary or metastatic tumors into blood circulation and act as the origin of distant metastasis [10]. Many studies have reported that enumeration of CTCs is significant to evaluate a prognosis, predict therapy efficacy, and monitor recurrence or metastasis in various malignancies including breast, colorectal, and lung cancers, etc. Moreover, circulating tumor microemboli (CTM, a cluster of 2 or more CTCs) was also demonstrated to be contributory to tumor metastasis in various cancer types, even more malignant and aggressive than CTCs. CTCs and CTM have attracted increasing attention due to the development of detection technologies. A recent study performed by a strategy of integrated subtraction enrichment and immunostaining fluorescence in situ hybridization (SE-iFISH) had described that CTCs karyotyping upon ploidy of chromosome 8 may provide an approach for predicting chemotherapy response and monitoring chemo-resistance in advanced gastric cancer patients. Ge et al. recently even used this system to detect CTM in patients with glioma [11–17]. However, the role of CTCs and CTM with respect to diagnosis and treatment in NPC are unknown.

SE-iFISH enables non-hemolytic elimination of red blood cells (RBCs) and depletion of white blood cells (WBCs) by anti-multiple WBC antibody, and characterizes CTCs integrated phenotyping and karyotyping of centromere probe 8 (CEP8) regardless of epithelial cell adhesion molecule (EpCAM) and cytokeratins (CKs) expression on the tumor cell surface [17, 18]. In the present study, we applied SE-iFISH to detect and characterize CTCs and CTM in patients with NPC, and then we explored the relationship between CTCs number and clinical staging. In addition, the value of CTCs number and karyotyping to therapeutic efficacy in NPC was investigated. Furthermore, we explored the correlation between CTCs count and plasma EBV DNA level in NPC.

## Methods

### Patients and sample collection

Ethical Committee of West China Hospital of Sichuan University approved the study. Fifty consecutive patients with biopsy-proven nasopharyngeal carcinoma (NPC) were enrolled from December 2015 to June 2017. Patients with a history of other tumors were excluded. Consent forms in writing were obtained from all subjects. Clinical data were collected for gender, age, diagnosis, pathology type, TNM stage (staging for patients with relapse or distant metastasis referred to TNM stage at NPC initial diagnosis), and plasma EBV DNA level. All patients completed an evaluation before treatment including fiberoptic nasopharyngoscopy, nasopharyngeal and neck magnetic resonance imaging (MRI), chest computed tomography (CT), abdominal ultrasonography and a single photon emission computed tomography (SPECT) whole-body bone scan. Staging of NPC was performed according to the American Joint Committee on Cancer (AJCC) 2010. Abdomen MRI was performed when abdominal ultrasonography suggested the presence of liver metastasis. Twelve NPC patients were subjected to the first-line chemotherapy based on gemcitabine plus cisplatin. After 2 to 4 cycles of chemotherapy, clinical response evaluation was performed according to the Response Evaluation Criteria in Solid Tumors 1.1. Peripheral venous blood (6.0 ml) from each subject was collected into customized tubes containing Acid Citrate Dextrose (ACD)-anticoagulant (Becton Dickinson, Franklin Lakes, NJ, USA) after discarding the first 2.0 ml blood to avoid potential pollutant with skin epithelial cells. All samples were stored at room temperature until been processed within 24 h after collection [19].

### Detection of CTCs by SE-iFISH

Experiment was performed similarly to that published studies and according to the kit instruction (Cytelligen, San Diego, CA, USA) [15, 18]. Briefly, 6.0 ml peripheral venous blood was centrifuged at 800×g for 7 min at room temperature, then, removed supernatants above red blood cells to deplete serum. The left components were thoroughly mixed and overlaid on 3 ml hCTC Separation Matrix. Solutions were centrifuged at 450×g for 6 min at room temperature and white buffy above red blood cells was removed to another centrifuge tube. The collected white buffy was incubated with 150 μl immunomagnetic particles conjugated to anti-leukocytes monoclonal antibodies including anti-CD45 at room temperature for 20 min with gentle shaking. The solution was overlaid on 3.0 ml of hCTC separation matrix, then, centrifuged at 450×g for 5 min at room temperature. Supernatants above magnetic beads were collected and were subsequently subjected to magnetic separation using a magnetic stand (Cytelligen) to remove leukocytes. The bead-free solution was washed with 1 × CRC solution (Cytelligen) and then spun down twice at 700×g for 3 min, 650×g for 3 min at room temperature, separately. Sedimented cells were washed once with 1 × CRC solution (Cytelligen) and centrifuged at 1050×g for 3 min, followed by discarding supernatant. Cell pellets were incubated with 200 μl Antibody Preparation Solution-1, 1 μl Alexa Fluor 594-conjugated monoclonal anti-CD45 and 1 μl Alexa Fluor 488-conjugated anti-EpCAM (Cytelligen) at 30 °C for 20 min in the dark. Sedimented cells were washed once and subsequently centrifuged at 950×g for 3 min at room temperature. The sedimented cells then were thoroughly mixed with cell fixative and applied onto the coated CTC slides (Cytelligen). The slides underwent drying process at 32 °C for 10 h for subsequent iFISH analysis. The slides were soaked in the mixed solution including FR1 and FR2 solution (Cytelligen) at 37 °C for 10 min. Later, the slides followed by 1 × FR3 soaking at 27 °C for 2 min and dehydrated in 100% ethanol for 2 min. Centromere Probe 8 (CEP8) SpectrumOrange (Vysis, Abbott Laboratories, Abbott Park, IL, USA) was denatured at 76 °C for 10 min and hybridized at 37 °C for 4 h. Finally, the slides were soaked in 1 × FR3 solution (Cytelligen) for 2 min and washed once, following stained with 4′,6-diamidino-2-phenylindole (DAPI, Vector laboratories, CA, USA) and subsequently subjected to fluorescence microscope (Carl Zeiss, Jena, Germany).

### Statistical analysis

All patients′ data were collected in an Excel database including clinicopathologic parameters such as patient ID, age, gender, diagnosis, pathology type, clinical stage, treatment procedure, and plasma EBV DNA level, then, merged with the CTCs enumeration, CTM enumeration and the phenotyping and karyotyping of CTCs at final analysis. Number was expressed as mean ± standard deviation (SD), and all statistical analyses were performed with SPSS 21.0 software. Comparison of CTCs number between two groups was done with independent sample t test. Correlation between CTCs numbers and EBV DNA level of NPC patients was assessed by Pearson linear correlation test. *P* < 0.05 was considered statistically significant. All the tests were two-sided.

## Results

### Patient characteristics

From December 2015 to June 2017, a total of 50 patients with confirmed nasopharyngeal carcinoma including twenty-nine newly diagnosed patients without distant metastasis (M0) and twenty-one relapsed or distant metastatic patients after 6 months of initial treatment were enrolled in this study. The median age of all patients was 48 (range from 17 to 66) years. Forty-nine patients were the World Health Organization (WHO) II-type or III-type, and only one patient was the WHO I-type. Among the 29 newly diagnosed (M0) patients, 93.1% (27/29) of patients were classified as stage III/IV. No patient with stage I was enrolled. Clinical characteristics of 50 patients were shown in Table 1.

**Table 1:** Characteristics of 50 NPC patients

Characteristics	Total	No. of patients	
		Newly diagnosis (M0, *N* = 29)	Relapse/Distant metastasis after treatment (*N* = 21)
Gender			
Male	34	19	15
Female	16	10	6
Age (years)			
Median (Range)	48 (17–66)	47 (17–66)	49(20–66)
< 50	29	17	12
≥ 50	21	12	9
Pathology type			
WHO I	1	1	0
WHO II/III	49	28	21
Stage^a^			
T-classification			
T1	7	5	2
T2	8	5	3
T3	13	7	6
T4	22	12	10
N-classification			
N0	4	3	1
N1	8	1	7
N2	29	20	9
N3	9	5	4
TNM stage			
I	0	0	0
II	4	2	2
III	19	12	7
IV	27	15	12
Treatment^b^			
IndCT+RT/CCRT	29	29	0
CT ± RT	15	0	15
Others	6	0	6
pEBV DNA level			
Negative	7	2	5
Positive	43	27	16

^a^ Stage for patients with relapse/distant metastasis after treatment refered to TNM stage at NPC initial diagnosis

^b^ *IndCT* = induction chemotherapy, *CT* = Chemotherapy, *RT* = radiotherapy, *CCRT* = concurrent chemoradiotherapy

### Identification of CTCs and CTM of NPC patients

**Fig. 1:**
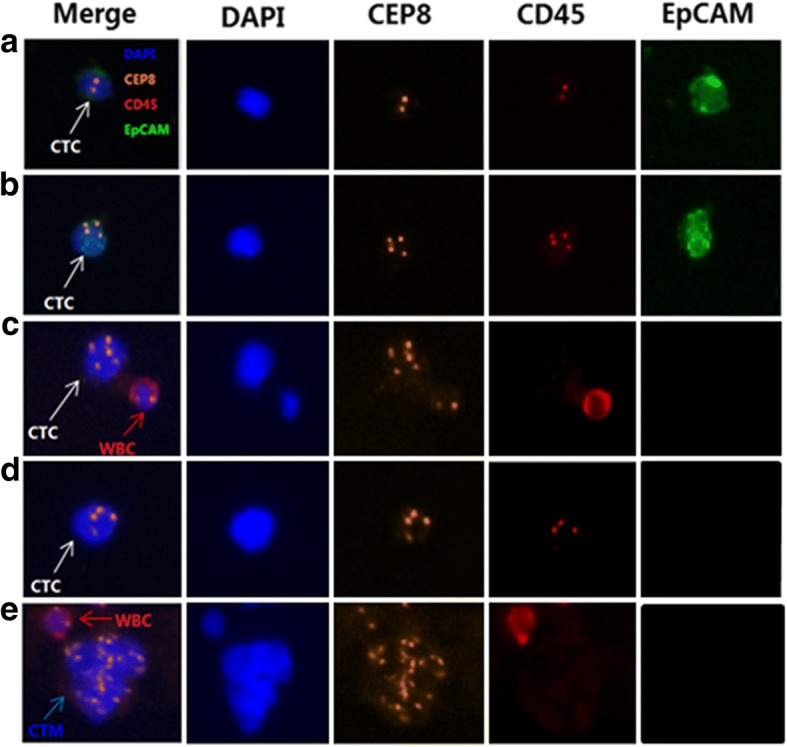
Identification of CTCs and CTM in NPC by SE-iFISH system. DAPI: blue, CEP8: orange, CD45: red, EpCAM: green. **a**. DAPI+/CD45-/EpCAM+/CEP8 = 2 (white arrow); **b**. DAPI+/CD45-/EpCAM+/CEP8 > 2 (white arrow); **c** DAPI+/CD45-/EpCAM-/CEP8 > 2 (white arrow), DAPI+/CD45+/EpCAM-/CEP8 = 2 (red arrow); **d**. DAPI+/CD45-/EpCAM-/CEP8 > 2 (white arrow); **e**. a cluster of CTCs (blue arrow)

All patients′ blood samples were subjected to subtraction enrichment (SE), following by EpCAM-iFISH analysis. We were inspired by previous studies in other cancer types. CTCs were identified as nucleated cells (DAPI+) with epithelial markers (EpCAM+) and/or aneuploidy (CEP8+) but without WBC marker (CD45-) [15, 18, 19]. Specifically, CTCs were defined as EpCAM+/CD45-/DAPI+/CEP8 ≥ 2, EpCAM-/CD45-/DAPI+/CEP8 > 2. Nucleated cells with CD45+ was defined as WBC. CTM was defined as a cluster of 2 or more CTCs (Fig. 1).

### The relationship between positive rate of CTCs and clinical characteristics

According to above definition, CTCs were detected in 46 cases (92.0%) among the 50 NPC patients, and CTM were observed in 6 patients (12.0%) in this study. The positive rate of CTCs in newly diagnosed (M0) patients and relapsed or distant metastatic patients was 93.1% (27/29) and 90.5% (19/21), respectively. CTM positive rate was 10.3% (3/29) and 14.3% (3/21) in these two groups of patients separately. No significant association was observed between CTCs positive rate and clinical parameters including gender, age, pathological type, and TNM stage (Table 2).

**Table 2:** The relationship between positive rate of CTCs and clinical characteristics

Characteristics	No. of patients with positive CTCs (%)	
	Newly diagnosis (M0, N = 29)	Relapse/Distant metastasis after treatment (*N* = 21)
Gender		
Male	17 (89.5)	14 (93.3)
Female	10 (100.0)	5 (83.3)
Age (years)		
< 50	16 (94.1)	11 (91.7)
≥ 50	11 (91.7)	8 (88.9)
Pathology type		
WHO I	1 (100.0)	–
WHO II/III	26 (92.9)	19 (90.5)
TNM stage^a^		
I	–	–
II	1 (50.0)	2 (100.0)
III	11 (91.7)	6 (85.7)
IV	15 (100.0)	11 (91.7)

^a^ Stage for patients with relapse/distant metastasis after treatment refered to TNM stage at NPC initial diagnosis

### Analysis of CTCs number and disease staging on NPC patients

In the present study, CTCs number per 6.0 ml peripheral blood in 50 patients was 0–45 (mean ± SD, 5.8 ± 9.5). CTCs number was 0–24 CTCs/6.0 ml (mean ± SD, 3.7 ± 4.6 CTCs/6.0 ml) in the 29 newly diagnosed patients without distant metastasis (M0) and 0–45 CTCs/6.0 ml (mean ± SD, 8.6 ± 13.3 CTCs/6.0 ml) in the 21 relapsed or distant metastatic patients. CTCs number in relapsed/distant metastatic patients was more than in newly diagnosed (M0) patients. This difference was no statistically significance (*P* = 0.117). TNM stage was an important clinical factor for NPC patients. To evaluate the correlation of CTCs number and clinical stage in NPC, we compared the relationship between CTCs number and T-classification, N-classification and TNM stage in this study (Table 3). In the newly diagnosed (M0) patients, CTCs number per 6.0 ml peripheral blood in stage II/III/IV were 0.5 ± 0.7 (range, 0–1), 3.7 ± 3.4 (range, 0–13), and 4.1 ± 5.7 (range, 1–24), respectively. Similarly, CTCs number of relapsed or distant metastatic patients in stage II/III/IV were 2.5 ± 2.1 (range, 1–4), 8.6 ± 13.3 (range, 0–12), and 12.0 ± 16.8 (range, 0–45), respectively. Staging of relapsed/distant metastatic patients referred to TNM stage at NPC initial diagnosis. CTCs number constantly increased with TNM stage rising (from stage II to stage IV) no matter in newly diagnosed (M0) patients and relapsed/distant metastatic patients. Nevertheless, CTCs number was not significantly associated with T-classification or N-classification.

**Table 3:** The relationship between CTCs enumeration and TNM stage of NPC patients

	Newly diagnosis (M0, *N* = 29)			Relapse/Distant metastasis after treatment (N = 21)		
Stage^a^	N^b^	No. of CTCs		N^b^	No. of CTCs	
		Range	Mean ± SD		Range	Mean ± SD
Total	29	0–24	3.7 ± 4.6	21	0–45	8.6 ± 13.3
T-classification						
T1	5	1–3	2.0 ± 1.0	2	1–4	2.5 ± 2.1
T2	5	0–13	3.8 ± 5.3	3	1–8	3.3 ± 4.0
T3	7	0–7	3.0 ± 2.2	6	0–22	3.0 ± 3.0
T4	12	1–24	4.8 ± 6.2	10	0–45	12.1 ± 17.9
N-classification						
N0	3	0–5	3.0 ± 2.6	1	16	16.0
N1	1	1	1.0	7	1–45	16.4 ± 19.8
N2	20	0–24	4.4 ± 5.4	9	0–8	2.6 ± 2.6
N3	5	1–3	2.0 ± 0.7	4	1–22	6.8 ± 10.1
TNM stage						
I	0	–	–	0	–	–
II	2	0–1	0.5 ± 0.7	2	1–4	2.5 ± 2.1
III	12	0–13	3.7 ± 3.4	7	0–12	8.6 ± 13.3
IV	15	1–24	4.1 ± 5.7	12	0–45	12.0 ± 16.8

Detectable CTCs were existence in 27 newly diagnosed (M0) patients and 19

relapsed/distant metastatic patients. Data were presented as mean ± standard deviation

^a^ Stage for patients with relapse/distant metastasis after treatment refered to TNM stage at NPC initial diagnosis

^b^ N referred to number of patients

### Correlation of CTCs number to therapeutic efficacy

**Fig. 2:**
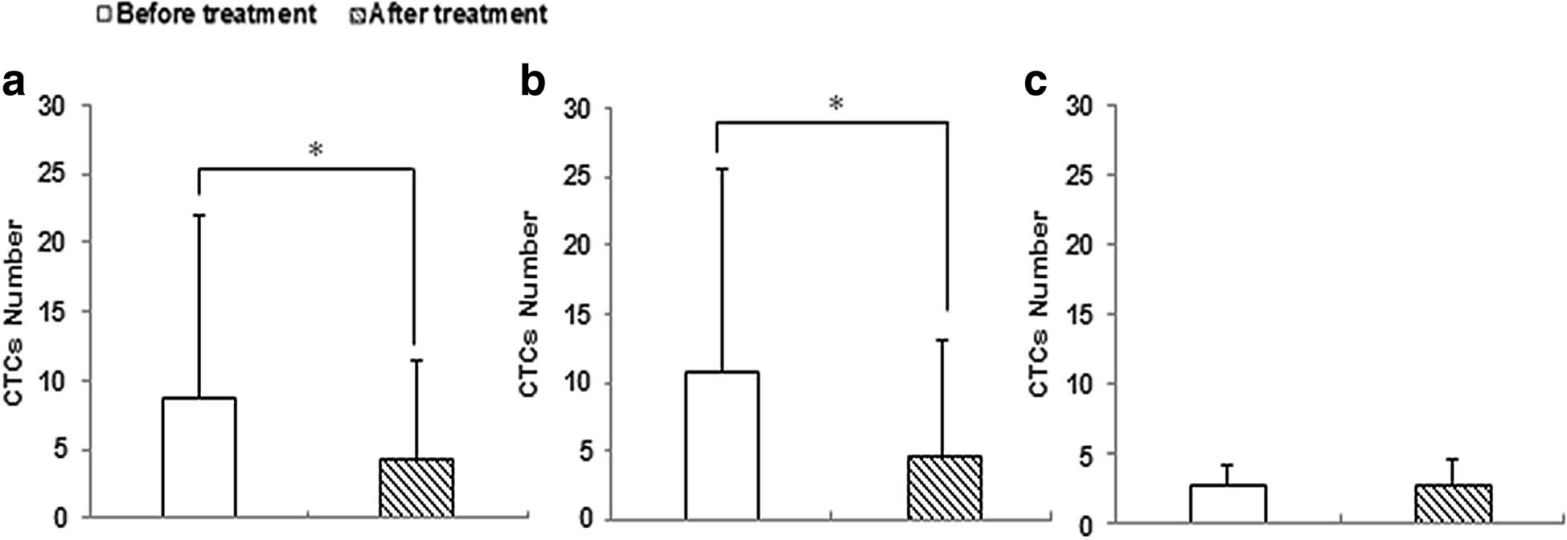
The relationship between CTCs number and therapeutic efficacy in NPC. **a**. CTCs number in 12 NPC patients decreased from 8.8 ± 13.1 (before treatment) to 4.3 ± 7.2 (after treatment) following 2 to 4 cycles of chemotherapy. **b**. CTCs number in patients with PR decreased from 10.8 ± 14.8 (before treatment) to 4.7 ± 8.3 (after treatment). **c**. CTCs number in PD/SD patients increased from 2.7 ± 1.5 (before treatment) to 3.0 ± 1.7 (after treatment). * *P* < 0.05

The changes of CTCs number were investigated in the 12 NPC patients who underwent the first-line gemcitabine plus cisplatin chemotherapy, including 7 newly diagnosed (M0) patients and 5 relapsed or distant metastatic patients. Correlation of CTCs number to therapeutic efficacy was shown in Fig. 2a. After 2 to 4 cycles of chemotherapy, the number of CTCs decreased from 8.8 ± 13.1 (range, 1–45) to 4.3 ± 7.2 (range, 0–26), and the difference was statistically significant (*P* = 0.049). Additional analysis was performed on those 12 cases who were subdivided into 2 groups based on their responses to treatment: partial response (PR, 30% or more decrease in the sum of diameters of target lesions), and progressive disease or stable disease (PD, at least 20% increase in the sum of diameters of target lesions; SD, less 30% decrease and less 20% increase in the sum of diameters of target lesions). CTCs number in PR patients dramatically decreased from 10.8 ± 14.8 (range, 1–45) to 4.7 ± 8.3 (range, 0–26) following treatment, with statistically significance (*P* = 0.040), as shown in Fig. 2b. Whereas, instead of decrease, CTCs number in PD/SD patients even slightly increased from 2.7 ± 1.5 (range, 1–4) to 3.0 ± 1.7 (range, 2–5) after chemotherapy. It was also noticed that CTCs were disappeared from 2 patients with PR following chemotherapy. Obtained results suggested that alterations of CTCs number were correlation to treatment responses, and monitoring CTCs number could predict therapeutic efficacy.

### Analysis of ploidy of chromosome 8 in CTCs of NPC patients

**Fig. 3:**
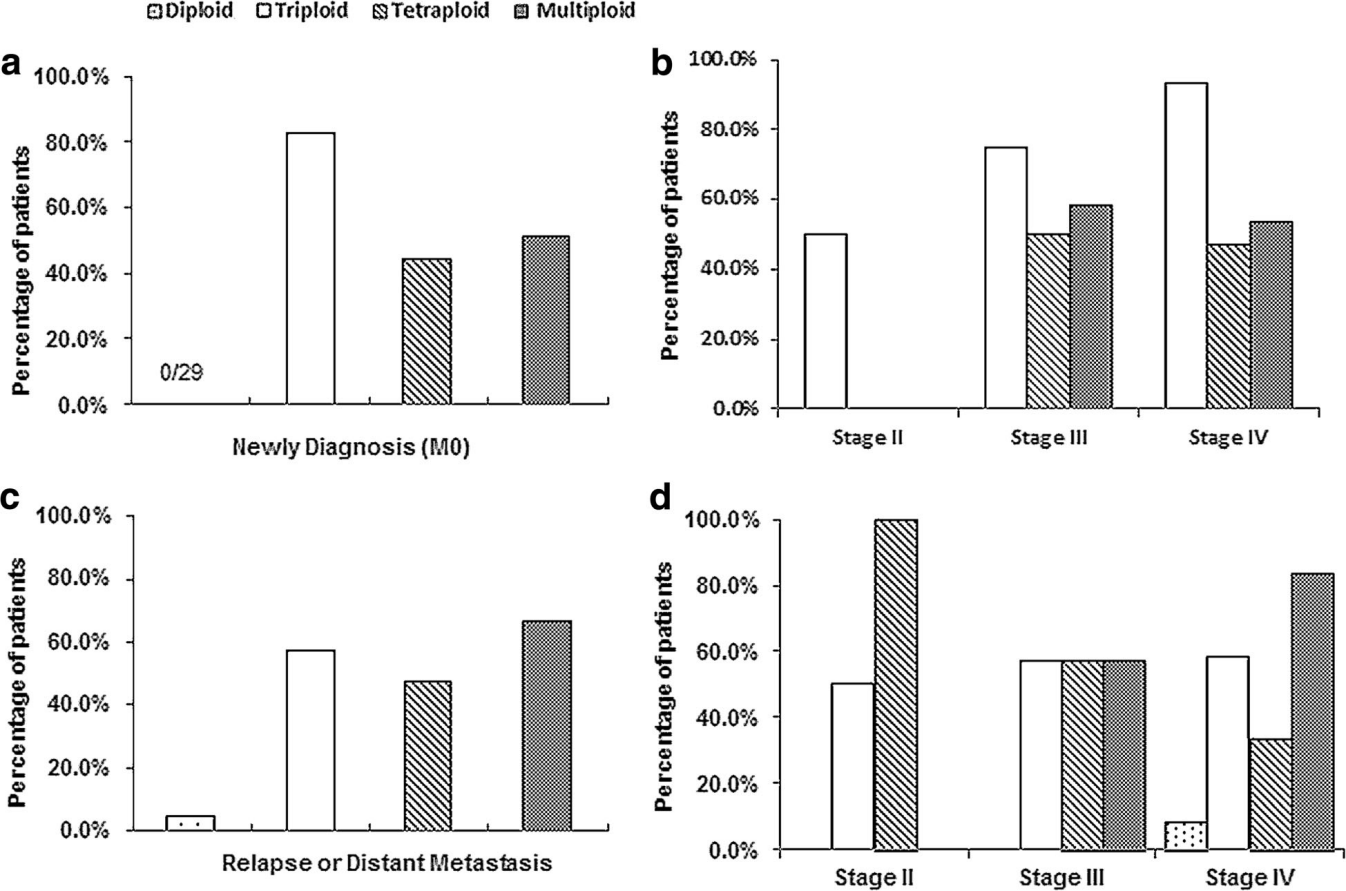
Correlation of ploidy of chromosome 8 in CTCs and disease staging. **a**. The ratio of CTCs with different karyotypes were 82.8% (triploidy), 44.8% (tetraploidy) and 48.3% (multiploidy) in newly diagnosed (M0) patients. **b**. For newly diagnosed (M0) patients, the frequency of triploid CTCs in stage II/III/IV were 50.0, 75.0, and 93.3%, respectively. Tetraploid CTCs ratio in stage II/III/IV were 0, 50.0, 46.7%. Multiploid CTCs ratio in stage II/III/IV were 0, 58.3, 53.3%. **c**. The ratio of CTCs with different karyotypes were 4.8% (diploidy), 57.1% (triploidy), 47.6% (tetraploidy) and 66.7% (multiploidy) in patients with relapse or distant metastasis. **d**. For patients with relapse or distant metastasis, the frequency of triploid CTCs in stage II/III/IV were 50.0, 57.0 and 58.3%, respectively. Tetraploid CTCs ratio in stage II/III/IV were 100, 57.0, 33.3%. Multiploid CTCs ratio in stage II/III/IV were 0, 57.0, 83.3%. Diploid CTCs was detected in only one patient. Multiploidy contains pentaploidy and those > 5 copies of chromosome 8 CTCs. Stage for patients with relapse/distant metastasis after treatment refered to TNM stage at NPC initial diagnosis

Based on the in situ karyotyping of SE-iFISH, aneuploidy of chromosome 8 in CTCs of NPC patients prior to treatment was examined. We evaluated the relationship between CTCs karyotyping and disease staging. In this study, diploid, triploid, tetraploid, and multiploid (pentaploid or > 5 copies of chromosome 8) CTCs were observed in the 50 NPC patients. The ratio of CTCs with different ploids of chromosome 8 were 2.0% (diploidy), 72.0% (triploidy), 46.0% (tetraploidy) and 58.0% (multiploidy) in all pre-treatment patients. For 29 newly diagnosed (M0) patients, the ratio of CTCs with different karyotypes were 82.8% (triploidy), 44.8% (tetraploidy) and 48.3% (multiploidy), while no diploid CTCs was found in those patients, as shown in Fig. 3a. Relationship between CTCs karyotyping and disease staging was further analysed. Results indicated that triploidy was the largest comparing with other karyotypes of CTCs in newly diagnosed (M0) patients, and the ratio of triploid CTCs were 50.0, 75.0, and 93.3% in stage II/III/IV of those patients, respectively (Fig. 3b). However, the frequency of CTCs with different karyotypes in 21 relapsed or distant metastatic patients were 4.8% (diploidy), 57.1% (triploidy), 47.6% (tetraploidy) and 66.7% (multiploidy), shown in Fig. 3c. Noticeably, the percentage of multiploid CTCs was the most in patients with relapse or distant metastasis. Furthermore, the ratio of multiploid CTCs was increasing in later stage patients with relapse or distant metastasis (Fig. 3d). We also studied the relationship between CTCs number with different karyotypes and disease staging in NPC, shown in Table 4. Triploid CTCs number was the most in newly diagnosed (M0) patients, while multiploid CTCs was the most in patients with relapse or distant metastasis. No significant difference was revealed between CTCs number with different karyotypes and TNM stage in NPC.

**Table 4:** The relationship between CTCs number with different karyotypes and TNM stage of NPC patients

Stage^a^	Newly diagnosis (M0, *N* = 29)			Relapse/Distant metastasis after treatment (*N* = 21)			
	Mean number of CTCs			Mean number of CTCs			
	Triploid	Tetraploid	Multiploid^b^	Diploid^c^	Triploid	Tetraploid	Multiploid
Total	1.6	0.5	1.1	1.2	2.1	1.6	3.7
T-classification							
T1	1.5	0.25	0.5	0	1.0	1.5	0
T2	1.2	0.6	2.0	0	1.7	0.3	1.3
T3	1.3	0.7	1.0	0	3.0	2.0	2.5
T4	2.1	1.1	1.6	2.6	2.0	1.7	5.8
N-classification							
N0	1.7	1.0	0.3	0	8.0	7.0	1.0
N1	1.0	0	0	3.7	2.6	2.7	7.4
N2	1.7	0.8	1.9	0	1.7	0.2	0.7
N3	1.4	0.6	0	0	1.0	1.3	4.5
TNM stage							
II	0.5	0	0	0	1.0	1.5	0
III	1.5	0.6	1.6	0	2.7	1.1	0.7
IV	1.9	1.0	1.3	2.2	2.0	1.8	6.0

^a^ Stage for patients with relapse/distant metastasis after treatment refered to TNM stage at NPC initial diagnosis

^b^ Multiploid of chromosome 8 contained pentaploidy and those > 5 copies

c Only one patient with distant metastasis was detected EpCAM (+) diploid CTCs

### Correlation of ploidy of chromosome 8 in CTCs to therapeutic efficacy

**Fig. 4:**
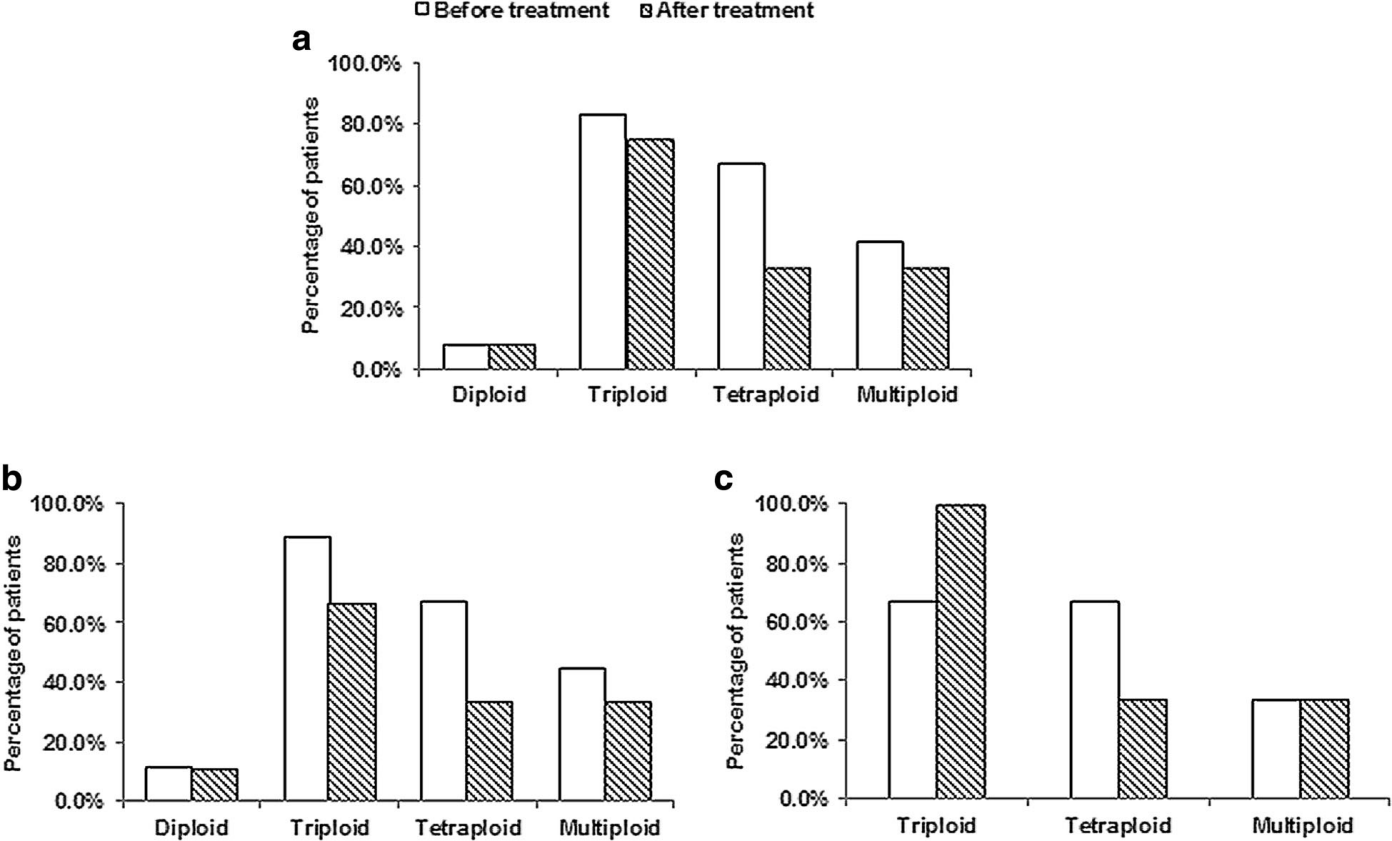
Comparison of CTCs karyotyping before and after treatment. **a**. The ratio of triploid, tetraploid, and multiploid CTCs in 12 NPC patients decreased after chemotherapy, while diploid CTCs was remained the same. **b**. For PR patients, the ratio of tetraploid CTCs definitely decreased from 66.7 to 33.3%, while triploid CTCs decreased from 88.9 to 66.7%, and multiploid CTCs decreased from 44.4 to 33.3% after treatment. **c**. For PD/SD patients, the frequency of tetraploid CTCs decreased from 66.7 to 33.3%, while triploid CTCs increased from 66.7 to 100%, and multiploid CTCs remained unchanged (33.3% versus 33.3%) after treatment

We monitored the changes of CTCs karyotyping of the 12 NPC patients post to chemotherapy based on gemcitabine plus cisplatin. For those patients prior to treatment, the ratio of CTCs with different chromosome 8 copy numbers were 8.3% (diploidy), 83.3% (triploidy), 66.7% (tetraploidy) and 41.7% (multiploidy), shown in Fig. 4a. Significantly decreased frequency was observed in tetraploid CTCs (66.7 to 33.3%), whereas decreased frequency of both triploidy (83.3 to 75.0%) and multiploidy (41.7 to 33.3%) was lower than tetraploidy. In addition, the frequency of diploid CTCs remained the same (8.3% versus 8.3%) after treatment. Additional analysis was performed on those 12 patients according to their responses to treatment, shown in Fig. 4b and c. The ratio of triploid CTCs decreased from 88.9% (before treatment) to 66.7% (post treatment) in PR patients, however, increased from 66.7.0% (before treatment) to 100% (post treatment) in PD/SD patients. For tetraploid CTCs, the ratio decreased from 66.7% (before treatment) to 33.3% (post treatment) in PR patients, and the same result was observed in PD/SD patients, from 66.7% (before treatment) to 33.3% (post treatment). In addition, the ratio of multiploid CTCs in PR patients was also decreased from 44.4% (before treatment) to 33.3% (post treatment), but still remained the same (33.3% versus 33.3%) in PD/SD patients after chemotherapy. In summary, following gemcitabine plus cisplatin chemotherapy, the ratio of tetraploid CTCs decreased obviously in all of 12 patients. Nevertheless, the ratio of triploid CTCs just decreased slightly in PR patients, but increased significantly in PD/SD patients. And the percentage of multiploid CTCs decreased in PR patients, but remained the same in PD/SD patients. Noticeably, those patients with PD/SD were relapsed/distant metastatic NPC patients. Results suggested that unlike sensitivity to chemotherapy for tetraploid CTCs, triploid and multiploid CTCs might be susceptible to drug resistance. Triploid and multiploid CTCs may correlate to chemo-resistance in relapsed/distant metastatic NPC patients.

### The relationship between CTCs count and plasma EBV DNA level on NPC patients

**Fig. 5:**
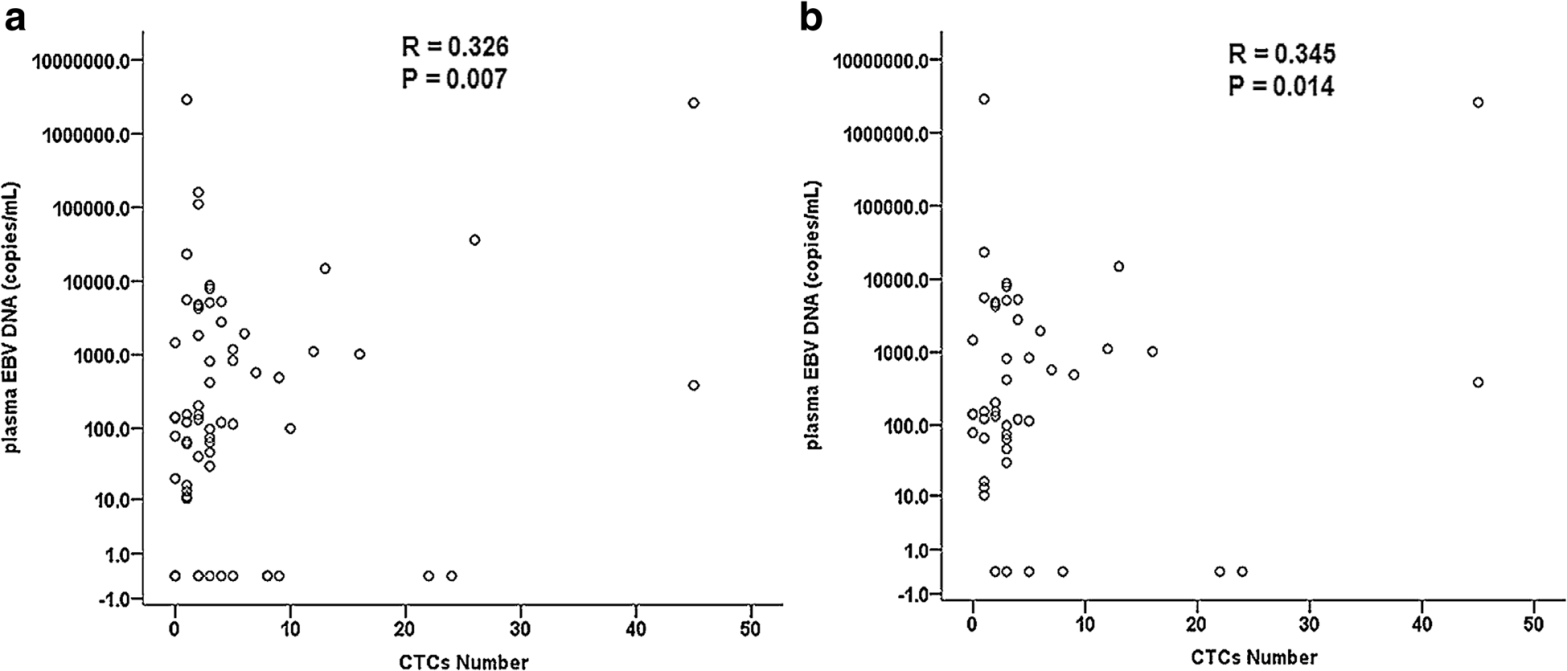
The relationship between CTCs number and plasma EBV DNA level of NPC patients. **a**. Positive correlation between CTCs number and plasma EBV DNA level was found in the 67 tested samples including 50 patients prior to treatment and 17 patients post to treatment (*R* = 0.326, *P* = 0.007). **b**. Positive correlation between CTCs number and plasma EBV DNA level in 50 pre-treatment patients was found (*R* = 0.345, *P* = 0.014). R, Pearson linear correlation coefficient; P, *P* value

NPC has been proven as an EBV-associated carcinoma, and most previous studies have established that plasma EBV DNA level is a significant prediction marker of distant metastasis in NPC [20, 21]. We evaluated the relationship between CTCs number and plasma EBV DNA level. Among the 67 tested samples including 50 pre-treatment and 17 post-treatment patients, the positive rate of plasma EBV DNA was 80.6% (54/67), and CTCs positive rate was 88.1% (59/67). Slight positive correlation was found between CTCs count and plasma EBV DNA level. The Pearson linear correlation coefficient (R) between them was 0.326, and the association was statistically significant (*P* = 0.007), shown in Fig. 5a. For 50 NPC patients prior to treatment, the positive rate of plasma EBV DNA was 86.0% (43/50), CTCs positive rate was 92.0% (46/50). Additional analysis about the 50 pre-treatment samples of NPC revealed that positive correlation was also found between CTCs number and plasma EBV DNA level prior to treatment, with statistically significant (*R* = 0.345, *P* = 0.014), shown in Fig. 5b. Interestingly, seven pre-treatment patients with negative EBV DNA level were patients with detectable CTCs, and 5 cases of those were confirmed as patients with relapse or distant metastasis. Results indicated that CTCs number in peripheral venous blood had a positive correlation with plasma EBV DNA level of NPC patients, and detecting CTCs by use of SE-iFISH was more sensitive than plasma EBV DNA level test.

## Discussion

Circulating tumor cells had been established as a risk factor of tumor progressions in various kinds of solid tumor types. It also has been reported that CTCs are promising to detect the harboring small tumors including primary and metastatic lesions in pancreatic cancer, while traditional imaging modalities fail to detect [22]. Recent studies even had demonstrated that CTCs might be an independent prognostic factor and CTCs presence seemed related to tumor burden. Moreover, changes of CTCs number had also been found to be correlation with treatment response to carcinomas of head and neck [23–27]. Si et al. reported that CTCs were tightly correlated with characteristics of NPC patients and CTCs number was decreased after therapy in NPC [28]. Nevertheless, the value of CTCs in the fields of diagnosis and treatment in NPC was still vague.

In this study, SE-iFISH automatic testing system was applied to detect CTCs and CTM in NPC. This strategy ensured the depletion of WBCs and removed RBCs effectively, and later, combined DAPE, CEP8, EpCAM and CD45 to identify CTCs. SE-iFISH platform improved the sensitivity of CTCs detection regardless of cancer heterogeneity, down-regulation or absence of CKs and EpCAM. In addition, aneuploidy of chromosome 8 detected by CEP8-FISH had been reported in gastric cancer, bladder cancer and lung cancer [18]. Through the previous description, the definition of CTCs in the present study were EpCAM+/CD45-/DAPI+/CEP8 ≥ 2, EpCAM -/CD45-/DAPI+/CEP8 > 2. A cluster of 2 or more CTCs defined as CTM. In this study, SE-iFISH yielded CTCs detection rate and CTM detection rate of 92.0 and 12.0% in NPC prior to treatment. Specifically, the CTCs and CTM positive rate of newly diagnosed (M0) patients were 93.1 and 10.3% respectively. While recent data presented by He et al. showed that CTCs/CTM was detected in 66.7 and 6.1% of NPC patients using ISET assay, separately, which were considerably lower than the detection rate in our study [28, 29].

In the current study, investigation of the association with CTCs positive rate and disease staging indicated that the detection rate of CTCs in newly diagnosed (M0) and relapsed/distant metastatic patients were consistent, and the difference of CTCs positive rates in different TNM stags was not significant. Reasons accounting for the results could be that 93.1% of newly diagnosed (M0) patients in this study were stage III/IV, on the one hand, lacking of patients with stage I. On the other hand, the presence of CTCs revealed the disease status during CTCs detection, and patients with relapse or distant metastasis did not certainly present CTCs in peripheral blood.

Investigation of CTCs counts in NPC was rarely reported. Our research in this paper indicated that CTCs number was related to clinical stage of NPC patients. CTCs number in patients with relapse or distant metastasis was much more comparing with newly diagnosed (M0) patients. Furthermore, the number of CTCs gradually increased in later stage patients of both newly diagnosis (M0) and relapse or distant metastasis. However, the T-classification or N-classification was not obviously related to CTCs number. Results showed that CTCs number could indicate the severity degree of disease and tumor burden in NPC. CTCs number revealed the common results of various factors of TNM classification.

Variations in CTCs number had been demonstrated association with the therapeutic efficacy in breast cancer, while current researches about the relationship between CTCs number and therapy in NPC were deficient [28, 30]. In this study, we observed that CTCs number was remarkably reduced in PR patients following 2 to 4 cycles of chemotherapy, on the contrary, CTCs number in PD/SD patients was slightly increased, and those patients with PD/SD were relapsed/distant metastatic patients. Obtained results demonstrated that changes of CTCs number were in accordance with responses to treatment of NPC patients, and the increase of CTCs number during treatment may indicate poor response to therapy. It is necessary to further expand the current study in order to evaluate CTCs as an indicator to be utilized to predict therapeutic efficacy and select optimal treatment protocols timely. Moreover, further studies are needed to explore the correlation of CTCs count and progression-free survival (PFS) or overall survival (OS) in NPC.

Aneuploidy of the chromosome exists in various tumor cells, and many studies showed that patients with aneuploid tumor cells had a worse outcome, that inspired us to investigate the aneuploidy of chromosome 8 in NPC [15, 31, 32]. In this study, analysis of CTCs karyotyping revealed that diploid, triploid, tetraploid, and multiploid CTCs were observed in NPC, indicating that the heterogeneity of chromosome 8 exist in CTCs. Triploid CTCs was the most comparing with other CTCs karyotypes in newly diagnosed patients without metastasis. Otherwise, the ratio of multiploid CTCs was the largest in relapsed/distant metastatic patients, in addition, multiploidy ratio was more in patients who were later-stage at initial diagnosis of NPC. Results indicated that multiploid CTCs might have higher malignant degree.

It has been recently reported that colorectal cancer cell lines with aneuploidy are associated with intrinsic resistance and have significantly worse clinical outcome [33]. Therefore, we investigated the correlation of CTCs karyotyping to chemotherapy efficacy of NPC patients in this study. Results demonstrated that CTCs with triploid and multiploid chromosome 8 were likely to correlate to the acquired resistance to chemotherapy based on gemcitabine plus cisplatin in relapsed/distant metastatic patients, rather than tetraploid CTCs performing initially sensitive to chemotherapy. It is also reasonable that karyotyping of CTCs could predict chemotherapy efficacy and monitor drug resistance. Similarly, Li et al. reported that triploid CTCs was related to intrinsic drug resistance, while tetraploid and multiploid CTCs were related to acquired drug resistance to paclitaxel or cisplatin-based chemotherapy in gastric cancer [15]. Reasons accounting for the poor concordance might be that chemo-sensitivity and drug-resistance of CTCs karyotyping in different carcinomas and drugs could have multiple mechanisms, and for cancers with effective treatment such as NPC might rarely display intrinsic drug resistance. Extended studies of karyotypic characterization of CTCs on NPC patients are underway.

Plasma EBV DNA level is a significant biomarker in monitoring relapse or distant metastasis in NPC, and the plasma EBV DNA level prior to treatment is correlated with tumor burden of NPC patients. The latest research declared that CTCs number was found to have positive correlation with EBV DNA load [29]. Currently, we compared the relationship between CTCs enumeration and plasma EBV DNA level in this study. Findings showed a positive correlation between CTCs number and plasma EBV DNA level in all NPC patients. Furthermore, the detection rate of CTCs in NPC by SE-iFISH was higher than the positive rate of plasma EBV DNA. CTCs could be used as a potential indicator for follow-up of NPC, and monitoring CTCs might be a supplementary method of discovering recurrence or metastasis in NPC.

In summary, NPC patients have a great survival rate for standard therapy, but distant metastasis and local recurrence were the main causes of treatment failure. CTCs are considered as the seeds of distant metastasis of malignant tumors. This study demonstrated blood testing of CTCs may be a prospective way in early diagnosis, treatment and follow-up of NPC. By detecting CTCs number and plasma EBV DNA level in combination, it is preferable to discover relapse and metastasis in the course of disease on NPC.

## Conclusions

In this study, SE-iFISH system could detect CTCs in NPC of various stages. Enumeration and karyotyping of CTCs in NPC might indicate the severity degree of disease, and dynamically monitoring CTCs number could evaluate therapeutic efficacy in real time. Analysis of CTCs karyotyping demonstrated that triploid and multiploid CTCs could play a significant role of drug resistance, therefore, karyotyping of CTCs may provide a potential approach for monitoring chemo-resistance and predicting chemotherapeutic efficacy in NPC. In addition, CTCs could be a biomarker during follow-up of NPC patients.

## Abbreviations

AJCC: American Joint Committee on Cancer
CCRT: Concurrent chemoradiotherapy
CEP8: Centromere probe 8
CKs: Cytokeratins
CT: Computed tomography
CTCs: Circulating tumor cells
CTM: Circulating tumor microemboli
EBV: Epstein-Barr virus
EpCAM: Epithelial cell adhesion molecule
IndCT: Induction chemotherapy
MRI: Magnetic resonance imaging
NPC: Nasopharyngeal carcinoma
OS: Overall survival
PD: Progressive disease
PFS: Progression-free survival
PR: Partial response
RBCs: Red blood cells
RT: Radiation therapy
SD: Stable disease
SD: Standard deviation
SE-iFISH: Subtraction enrichment and immunostaining fluorescence in situ hybridization
SPECT: Single photon emission computed tomography
WBCs: White blood cells
